# Evaluating the Causal Association Between Educational Attainment and Asthma Using a Mendelian Randomization Design

**DOI:** 10.3389/fgene.2021.716364

**Published:** 2021-08-09

**Authors:** Yunxia Li, Wenhao Chen, Shiyao Tian, Shuyue Xia, Biao Yang

**Affiliations:** ^1^Department of Respiratory and Critical Care Medicine, Affiliated Central Hospital, Shenyang Medical College, Shenyang, China; ^2^Department of Pathogen Biology, Shenyang Medical College, Shenyang, China

**Keywords:** asthma, educational attainment, genome-wide association study, Mendelian randomization, inverse-variance weighted

## Abstract

Asthma is a common chronic respiratory disease. In the past 10 years, genome-wide association study (GWAS) has been widely used to identify the common asthma genetic variants. Importantly, these publicly available asthma GWAS datasets provide important data support to investigate the causal association of kinds of risk factors with asthma by a Mendelian randomization (MR) design. It is known that socioeconomic status is associated with asthma. However, it remains unclear about the causal association between socioeconomic status and asthma. Here, we selected 162 independent educational attainment genetic variants as the potential instruments to evaluate the causal association between educational attainment and asthma using large-scale GWAS datasets of educational attainment (*n* = 405,072) and asthma (*n* = 30,810). We conducted a pleiotropy analysis using the MR-Egger intercept test and the MR pleiotropy residual sum and outlier (MR-PRESSO) test. We performed an MR analysis using inverse-variance weighted, weighted median, MR-Egger, and MR-PRESSO. The main analysis method inverse-variance weighted indicated that each 1 standard deviation increase in educational attainment (3.6 years) could reduce 35% asthma risk [odds ratio (OR) = 0.65, 95% confidence interval (CI) 0.51–0.85, *P* = 0.001]. Importantly, evidence from other MR methods further supported this finding, including weighted median (OR = 0.55, 95% CI 0.38–0.80, *P* = 0.001), MR-Egger (OR = 0.48, 95% CI 0.16–1.46, *P* = 0.198), and MR-PRESSO (OR = 0.65, 95% CI 0.51–0.85, *P* = 0.0015). Meanwhile, we provide evidence to support that educational attainment protects against asthma risk dependently on cognitive performance using multivariable MR analysis. In summary, we highlight the protective role of educational attainment against asthma. Our findings may have public health applications and deserve further investigation.

## Introduction

Asthma is a common chronic respiratory disease (Beasley et al., 2015; Han et al., 2020; von Mutius and Smits, 2020). It is estimated that asthma could affect over 300 million people in the world and result in a substantial burden (Beasley et al., 2015; Han et al., 2020; von Mutius and Smits, 2020). During the past 30 years, asthma death rates have decreased greatly (Beasley et al., 2015; von Mutius and Smits, 2020). However, there are still no effective therapeutic regimens (Beasley et al., 2015; von Mutius and Smits, 2020). Hence, it is important to identify the risk factors for asthma, especially those with the causation association for asthma (Beasley et al., 2015; von Mutius and Smits, 2020).

In the past 10 years, genome-wide association study (GWAS) has been widely used to identify the common asthma genetic variants (Demenais et al., 2018; Zhu et al., 2018; Shrine et al., 2019; Han et al., 2020). In 2018, the Trans-National Asthma Genetic Consortium (TAGC) conducted a GWAS analysis of asthma using 23,948 cases and 118,538 controls from kinds of populations, including European, African, Japanese, and Latino ancestries (von Mutius and Smits, 2020). They successfully found five new asthma loci (Demenais et al., 2018). Zhu et al. (2018) conducted a genome-wide cross-trait analysis of asthma and allergic diseases using large-scale GWAS datasets from the UK Biobank, including 33,593 cases and 76,768 controls of European ancestry. They found a significant genetic correlation between asthma and allergic diseases and highlighted 38 shared loci (Zhu et al., 2018). Shrine et al. (2019) carried out a GWAS analysis to identify common genetic variants associated with moderate-to-severe asthma by a two-stage design, including 5,135 asthma cases and 25,675 controls in stage 1 and 5,414 asthma cases and 21,471 controls in stage 2. Importantly, all these selected individuals are of European ancestry (Shrine et al., 2019). Interestingly, they reported 24 novel genetic variants to be significantly associated with moderate-to-severe asthma (Shrine et al., 2019). Han et al. (2020) conducted a GWAS analysis of asthma using 64,538 asthma cases and 329,321 controls from the UK Biobank. They further performed an asthma GWAS meta-analysis of the UK Biobank and the TAGC (Demenais et al., 2018; Han et al., 2020). Finally, Han et al. identified 66 novel asthma loci (Demenais et al., 2018; von Mutius and Smits, 2020).

Importantly, these publicly available asthma GWAS datasets provide important data support to investigate the causal association of kinds of risk factors with asthma by Mendelian randomization (MR) design or polygenic score (Granell et al., 2014; Minelli et al., 2018; Skaaby et al., 2018; Rosa et al., 2019; Xu et al., 2019; Zhao and Schooling, 2019; Chen et al., 2020; Mulugeta et al., 2020; Shen et al., 2020; Sun et al., 2020; Au Yeung et al., 2021a; Park et al., 2021; Raita et al., 2021). Some risk factors have been reported to increase the risk of asthma, including soluble interleukin-6 receptor level (Rosa et al., 2019; Raita et al., 2021), childhood body mass index (BMI) (Au Yeung et al., 2021a), adult BMI (Granell et al., 2014; Skaaby et al., 2018; Xu et al., 2019; Sun et al., 2020; Au Yeung et al., 2021a), major depressive disorder (Mulugeta et al., 2020), early pubertal maturation (Chen et al., 2020), and age at puberty (Minelli et al., 2018). Meanwhile, other risk factors are associated with reduced risk of asthma, including estimated glomerular filtration rate (Park et al., 2021), lifetime smoking (Shen et al., 2020), and linoleic acid (Zhao and Schooling, 2019).

In addition to these risk factors discussed earlier, socioeconomic status is also associated with asthma (Eagan et al., 2004; Hancox et al., 2004; Kozyrskyj et al., 2010; Brite et al., 2020). However, it remains unclear about the causal association between socioeconomic status and asthma (Eagan et al., 2004; Hancox et al., 2004; Kozyrskyj et al., 2010; Brite et al., 2020). Here, we selected 162 independent educational attainment genetic variants as the potential instruments to evaluate the causal association between educational attainment and asthma.

## Materials and Methods

### Educational Attainment Genome-Wide Association Study Dataset

We selected 162 independent genetic variants that influence educational attainment to be the potential instrumental variables (Okbay et al., 2016). In brief, these genetic variants are identified by a recent large-scale GWAS dataset of educational attainment in individuals of European descent (*n* = 405,072) (Okbay et al., 2016). The educational attainment was a continuous variable measuring by the number of years of schooling completed (EduYears) and was assessed at age or older than 30 years (Okbay et al., 2016). This large-scale GWAS dataset is based on the meta-analysis of GWAS results from the discovery stage (Social Science Genetic Association Consortium, including 293,723 individuals) and replication stage (UK Biobank, including 111,349 individuals) (Okbay et al., 2016). The participating cohorts in the discovery stage are provided in Table 1. Finally, this meta-analysis identified 162 independent genetic variants with the genome-wide significance (*P* < 5.00E-08), as provided in Supplementary Table 1 (Okbay et al., 2016).

**TABLE 1:** Participating cohorts in Educational attainment GWAS discovery stage (Okbay et al., 2016).

**Study**	**Country**	**Sample size**	**Birth year (mean/range)**	**Female %**
ACPRC	England	1,713	1923 (1903–1948)	0.71
AGES	Iceland	3,212	1927 (1908–1936)	0.58
ALSPAC	England	2,877	1959 (1948–1963)	1
ASPS	Austria	777	1932 (1909–1949)	0.57
BASE—II	Germany	1,619	1948 (1925–1983)	0.52
CoLaus	Switzerland	3,269	1950 (1928–1970)	0.53
COPSAC2000	Germany	318	1966 (1964–1969)	0.47
CROATIA—Korčula	Croatia	842	1950 (1909–1977)	0.64
deCODE	Iceland	46,758	1945 (1894–1983)	0.57
DHS	Germany	953	1949 (1929–1974)	0.53
DIL	England	2,578	1958 (1958–1958)	0.52
EGCUT1	Estonia	5,597	1950 (1905–1980)	0.55
EGCUT2	Estonia	1,328	1957 (1911–1979)	0.53
EGCUT3	Estonia	2,047	1966 (1930–1982)	0.73
ERF	Netherlands	2,433	1952 (1914–1974)	0.55
FamHS	United States	3,483	1941 (1900–1965)	0.53
FINRISK	Finland	1,685	1946 (1923–1977)	0.46
FTC	Finland	2,418	1945 (1910–1972)	0.56
GOYA	Denmark	1,459	1947 (1944–1954)	0
GRAPHIC	England	727	1951 (1942–1965)	0.53
GS	Scotland	8,776	1955 (1909–1981)	0.59
H2000 Cases	Finland	797	1949 (1924–1970)	0.5
H2000 Controls	Finland	819	1949 (1924–1969)	0.52
HBCS	Finland	1,617	1941 (1934–1944)	0.57
HCS	Australia	1,946	1940 (1920–1951)	0.49
HNRS (CorexB)	Germany	1,401	1942 (1926–1955)	0.5
HNRS (Oexpr)	Germany	1,347	1942 (1926–1955)	0.5
HNRS (Omni1)	Germany	778	1942 (1927–1955)	0.52
HRS	United States	9,963	1940 (1900–1979)	0.42
Hypergenes	Italy/United Kingdom/Belgium	815	1945 (1914–1971)	0.46
INGI—CARL	Italy	947	1946 (1910–1975)	0.58
INGI—FVG	Italy	943	1951 (1917–1978)	0.6
KORA S3	Germany	2,655	1945 (1920–1964)	0.51
KORA S4	Germany	2,721	1949 (1926–1970)	0.51
LBC1921	Scotland	515	1921 (1921–1921)	0.58
LBC1936	Scotland	1,003	1936 (1936–1936)	0.49
LifeLines	Netherlands	12,539	1960 (1921–1980)	0.58
MCTFR	United States	3,819	1953 (1926–1974)	0.54
MGS	United States	2313	1951 (1914–1976)	0.5
MoBa	Norway	622	1971 (1966–1976)	1
NBS	Netherlands	1,808	1941 (1923–1972)	0.5
NESDA	Netherlands	1,820	1958 (1939–1977)	0.64
NFBC66	Finland	5,297	1966 (1966–1966)	0.52
NTR	Netherlands	5,246	1958 (1917–1989)	0.64
OGP	Italy	370	1950 (1916–1976)	0
OGP—Talana	Italy	544	1949 (1910–1977)	0.59
ORCADES	Scotland	1,828	1952 (1914–1979)	0.6
PREVEND	Netherlands	3,578	1948 (1923–1968)	0.48
QIMR	Australia	8,006	1956 (1900–1984)	0.59
RS—I	Netherlands	6,108	1922 (1893–1938)	0.6
RS—II	Netherlands	1,667	1935 (1906–1944)	0.52
RS—III	Netherlands	3,040	1950 (1910–1960)	0.56
Rush—MAP	United States	887	1921 (1901–1948)	0.72
Rush—ROS	United States	808	1921 (1896–1946)	0.66
SardiNIA	Italy	5,616	1955 (1901–1983)	0.58
SHIP	Germany	3,556	1945 (1918–1971)	0.5
SHIP—TREND	Germany	901	1956 (1928–1980)	0.57
STR—Salty	Sweden	4,832	1951 (1943–1958)	0.52
STR—Twingene	Sweden	9,553	1941 (1916–1958)	0.53
THISEAS	Greece	829	1950 (1909–1979)	0.33
TwinsUK	England	4,012	1949 (1919–1978)	1
WTCCC58C	England	2,804	1958 (1958–1958)	0.48
YFS	Finland	2,029	1969 (1962–1977)	0.55
23andMe	Primarily US	76,155	1961 (1901–1985)	0.52

### Cognitive Performance Genome-Wide Association Study Dataset

We selected a large-scale GWAS dataset of cognitive performance in 257,841 individuals of European descent (Okbay et al., 2016). It is based on the sample-size-weighted meta-analysis of two large-scale GWAS datasets from the COGENT consortium (*n* = 35,298) and UK Biobank (*n* = 222,543) (Okbay et al., 2016). In COGENT, the phenotype measure was the first unrotated principal component of performance on at least three neuropsychological tests (or at least two IQ-test subscales) (Okbay et al., 2016). In the UK Biobank, the phenotype measure was a standardized score on a test of verbal–numerical reasoning (Okbay et al., 2016). More detailed information is provided in the original study (Okbay et al., 2016).

### Asthma Genome-Wide Association Study Dataset

We selected the large-scale asthma GWAS dataset in 30,810 individuals of European ancestry, including 5,135 moderate–severe asthma cases and 25,675 controls, as described in the original study (Shrine et al., 2019). These selected moderate–severe asthma cases are from the Genetics of Asthma Severity and Phenotypes study (GASP, *n* = 1,858), the Unbiased Biomarkers in Prediction of respiratory disease outcomes project (U-BIOPRED, *n* = 281), and the UK Biobank (*n* = 2,996) (Shrine et al., 2019). The selected controls are from the U-BIOPRED (*n* = 75) and the UK Biobank (*n* = 25,600) (Shrine et al., 2019). In GASP and U-BIOPRED, moderate-to-severe asthma patients were evaluated using clinical records based on the British Thoracic Society 2014 guidelines (Shrine et al., 2019). In the UK Biobank, moderate-to-severe asthma cases were diagnosed by a doctor (Shrine et al., 2019). The key demographic characteristics, including age and sex, are provided in Table 2 or the original study (Shrine et al., 2019).

**TABLE 2:** Baseline characteristics of asthma cases and controls (Shrine et al., 2019).

**Phenotypes**	**Cases (*n* = 5,135)**	**Controls (*n* = 25,675)**
Age, years	55 (12)	56 (8)
Female	3,170 (61.7%)	14,626 (57.0%)
Male	1,965 (38.3%)	11,049 (43.0%)
FEV*1*, % predicted	72.4% (21.4)	91.8% (17.4)
FEV*1*/FVC	0.67 (0.12)	0.76 (0.06)
Smoking status Ever smoker	2,265 (44.1%)	11,913 (46.4%)
Smoking status Never smoker	2,647 (51.6%)	13,487 (52.5%)
Smoking status Unknown	223 (4.3%)	275 (1.1%)
Rhinitis or eczema status Yes	1,897 (36.9%)	8^†^
Rhinitis or eczema status No	2,062 (40.2%)	25 667^†^
Rhinitis or eczema status Unknown	1,176 (22.9%)	0^†^
Rhinitis or eczema status Oral corticosteroid use (prednisolone)	222/3,710 (6.0%)	NA

*Data are mean (SD) or *n* (%), unless otherwise stated.*

*FEV1, forced expiratory volume in 1 s; FVC, forced vital capacity; NA, not applicable; U-BIOPRED, Unbiased biomarkers in prediction of respiratory disease outcomes.*

*^†^Patients in the U-BIOPRED cohort were not screened for rhinitis or eczema before sample selection but were subsequently found to comprise eight patients with rhinitis, eczema, or allergy.*

### Pleiotropy Analysis

MR is established based on three key assumptions. Assumption 1: genetic variants (instrumental variables) should be significantly associated with the exposure (educational attainment). Hence, we selected 162 independent genetic variants associated with educational attainment with the genome-wide significance (*P* < 5.00E-08), as described earlier. Both assumption 2 and assumption 3 are known as no pleiotropy, as described in recent MR studies (Larsson et al., 2020; Au Yeung et al., 2021b; Sun et al., 2021; Yuan et al., 2021; Zhao and Schooling, 2021; Zhuang et al., 2021b). Hence, we conducted a pleiotropy analysis using the MR-Egger intercept test (Bowden et al., 2015; Burgess and Thompson, 2017) and the MR pleiotropy residual sum and outlier (MR-PRESSO) test (Verbanck et al., 2018); both have widely used in recent MR studies (Larsson et al., 2020; Au Yeung et al., 2021b; Sun et al., 2021; Yuan et al., 2021; Zhao and Schooling, 2021; Zhuang et al., 2021b). The significance threshold *P* < 0.05 indicated evidence of pleiotropy.

### Mendelian Randomization Analysis

For univariable MR analysis, we selected the inverse-variance weighted (IVW) as the main MR analysis method. Meanwhile, we also selected other additional MR analysis methods, including weighted median, MR-Egger method, and MR-PRESSO, as used in recent MR studies (Bowden et al., 2015; Burgess and Thompson, 2017; Liu et al., 2018; Larsson et al., 2020; Au Yeung et al., 2021b; Sun et al., 2021; Yuan et al., 2021; Zhao and Schooling, 2021; Zhuang et al., 2021b). For multivariable MR analysis, we selected the multivariable IVW method, multivariable median-based method, and multivariable MR-Egger method. The odds ratio (OR) and 95% confidence interval (CI) of asthma correspond to approximately per 3.6 years increase [approximately 1 standard deviation (SD)] in EduYears. R (version x64 4.0.3), R package “MendelianRandomization,” and MR-PRESSO were used to perform the MR analysis. The significance threshold *P* < 0.05 indicated evidence of causal association. To test the influence of a single genetic variant, we also conducted a sensitivity analysis using leave-one-out permutation (Liu et al., 2018).

### Power Analysis

The variance of educational attainment (*R*^2^) explained by the selected genetic variants was calculated using the effect allele frequency, the effect size beta (*β*), and the number of the selected genetic variants (*k*), as described in a previous study (Locke et al., 2015).

R2=∑i=1kβi2(1-EAFi)*E*AFi2*

Based on the *R*^2^ and other necessary information, including sample size, type-I error rate, proportion of cases in the study, and true OR of the outcome variable per SD of the exposure variable, the statistical power was calculated using mRnd (Power calculations for MR) (Brion et al., 2013).

## Results

### Educational Attainment Genetic Variants and Asthma

We selected 162 independent genetic variants influencing educational attainment and extracted their corresponding summary statistics in the asthma GWAS dataset. The results showed that 141 unique genetic variants were available in the asthma GWAS dataset. Only five genetic variants are associated with asthma risk with *P* < 0.05, including rs1378214 (*P* = 0.000531), rs76878669 (*P* = 0.00607), rs7772172 (*P* = 0.00666), rs113520408 (*P* = 0.0183), and rs9556958 (*P* = 0.0388). These findings indicated that all these selected genetic variants showed a more significant trend associated with educational attainment. Table 3 provides the more detailed results about these 141 genetic variants.

**TABLE 3:** Association between 141 educational attainment genetic variants and asthma.

**SNP**	**CHR**	**Position (b37)**	**EA**	**NEA**	**EAF**	**Beta**	**SE**	***P*-value**
rs56044892	1	41830086	T	C	0.198	0.00536	0.0291	0.854
rs12076635	1	44026656	C	G	0.779	−0.0126	0.0265	0.634
rs12410444	1	44188719	G	A	0.297	−0.0303	0.0241	0.207
rs142328051	1	44371441	C	T	0.142	−0.00193	0.0289	0.947
rs2568955	1	72762169	C	T	0.739	−0.0225	0.0262	0.39
rs12142680	1	73615892	A	G	0.0757	0.018	0.0461	0.696
rs12145291	1	74161795	C	T	0.0546	−0.0294	0.053	0.579
rs1008078	1	91189731	T	C	0.397	0.0316	0.0226	0.163
rs12134151	1	96202443	C	G	0.494	0.00288	0.0219	0.895
rs4378243	1	98395881	T	G	0.834	−0.04	0.0299	0.182
rs17372140	1	98572382	A	G	0.295	0.0299	0.0244	0.22
rs648163	1	199315998	T	C	0.27	−0.00598	0.0247	0.809
rs11588857	1	204587047	A	G	0.205	0.0112	0.0272	0.681
rs35771425	1	211609768	C	T	0.221	−0.0371	0.0265	0.162
rs78365243	1	211737950	C	T	0.0499	0.058	0.0515	0.26
rs2992632	1	243503764	T	A	0.287	−0.00229	0.0248	0.927
rs7590368	2	10961474	C	T	0.257	0.0258	0.0251	0.305
rs76076331	2	10977585	T	C	0.123	0.016	0.0338	0.636
rs17504614	2	51080481	C	T	0.186	−0.0113	0.0286	0.692
rs56158183	2	60632924	A	G	0.0795	0.0447	0.0407	0.272
rs7593947	2	60704933	A	T	0.529	−0.0343	0.0226	0.129
rs356992	2	60753593	G	C	0.695	−0.0127	0.024	0.598
rs268134	2	65608363	G	A	0.752	−0.00901	0.0254	0.723
rs6715849	2	100306378	G	A	0.559	0.0105	0.0224	0.638
rs4851251	2	100753490	T	C	0.274	0.0216	0.0248	0.383
rs12987662	2	100821548	A	C	0.4	−0.0294	0.0225	0.191
rs71413877	2	100924822	A	G	0.0419	−0.0846	0.0558	0.13
rs34106693	2	101151830	G	C	0.171	0.0371	0.0301	0.218
rs77702819	2	101328728	T	G	0.0926	−0.0211	0.0388	0.587
rs17824247	2	144152539	C	T	0.41	−0.0298	0.0223	0.182
rs10178115	2	155451738	G	T	0.45	0.0216	0.0223	0.332
rs10930008	2	161854736	A	G	0.738	−0.0478	0.0249	0.0549
rs16845580	2	161920884	C	T	0.37	−0.00443	0.023	0.847
rs4500960	2	162818621	T	C	0.483	−0.0128	0.022	0.56
rs1596747	2	193802478	G	A	0.493	0.000776	0.0219	0.972
rs4675248	2	202880230	G	A	0.57	−0.0423	0.0229	0.0642
rs12694681	2	226609241	G	T	0.308	0.0044	0.0238	0.853
rs11687170	2	237058144	C	T	0.168	−0.0209	0.0293	0.475
rs7429990	3	47901803	A	C	0.275	0.00642	0.0245	0.793
rs140711597	3	48469441	G	C	0.0213	−0.0101	0.0823	0.902
rs34638686	3	48682658	T	C	0.0999	0.0572	0.0371	0.124
rs3172494	3	48731487	T	G	0.108	−0.0447	0.0357	0.211
rs113011189	3	49250007	T	C	0.0896	0.0311	0.039	0.424
rs13090388	3	49391082	T	C	0.303	−0.0368	0.0239	0.123
rs11130222	3	49901060	T	A	0.42	0.0279	0.0223	0.21
rs6800916	3	50052873	A	T	0.0953	−0.0565	0.0425	0.184
rs2624818	3	50056265	A	G	0.103	0.00892	0.0363	0.806
rs112634398	3	50075494	G	A	0.0487	0.0799	0.0527	0.13
rs71326918	3	50174844	A	C	0.116	−0.0201	0.0347	0.561
rs35971989	3	51469248	G	A	0.158	−0.00101	0.0306	0.974
rs7610856	3	71579022	A	C	0.43	−0.0393	0.0224	0.08
rs62263923	3	85674790	G	A	0.362	−0.0225	0.0231	0.33
rs56262138	3	86183716	A	T	0.299	0.00764	0.0245	0.755
rs9755467	3	127143885	T	C	0.159	−0.00189	0.0303	0.95
rs12646808	4	3249828	C	T	0.35	0.0203	0.0235	0.388
rs1967109	4	28720915	G	A	0.837	−0.000441	0.03	0.988
rs4308415	4	67821874	G	C	0.57	0.0253	0.022	0.25
rs6839705	4	106144735	C	A	0.662	0.0272	0.0232	0.241
rs4863692	4	140764124	T	G	0.325	0.011	0.0234	0.64
rs1912528	4	140945966	T	C	0.358	−0.00849	0.0229	0.71
rs12640626	4	176626272	A	G	0.568	−0.00204	0.0222	0.927
rs4493682	5	45188024	C	G	0.18	−0.0144	0.0287	0.615
rs1562242	5	57566494	C	T	0.516	0.0191	0.0221	0.388
rs61160187	5	60111579	G	A	0.403	0.0123	0.022	0.576
rs113474297	5	60554934	T	C	0.14	0.0511	0.0319	0.109
rs10223052	5	60800336	G	A	0.645	0.0181	0.0231	0.434
rs775326	5	62918416	A	C	0.322	0.0117	0.0234	0.617
rs12653396	5	87847273	A	T	0.568	−0.00266	0.0225	0.906
rs6882046	5	87968864	G	A	0.267	0.0359	0.025	0.151
rs700590	5	88106258	C	T	0.401	0.00243	0.0226	0.914
rs152603	5	106774922	G	A	0.361	0.00366	0.0229	0.873
rs660001	5	113866598	A	G	0.212	0.0201	0.0269	0.454
rs62379838	5	120102028	C	T	0.299	0.0269	0.024	0.263
rs7776010	6	14723608	C	T	0.19	−0.0418	0.0283	0.139
rs7772172	6	16662928	G	A	0.599	0.0609	0.0225	0.00666
rs6939294	6	16950631	T	C	0.228	−0.0092	0.0265	0.728
rs56231335	6	98187291	C	T	0.327	−0.0149	0.0235	0.525
rs1338554	6	98346801	G	A	0.504	−0.00034	0.0221	0.988
rs9401593	6	98549801	C	A	0.483	−0.0256	0.0222	0.25
rs56081191	6	98557732	A	G	0.0785	−0.00977	0.0418	0.815
rs11756123	6	152218079	T	A	0.633	−0.0368	0.0228	0.107
rs113779084	7	11871787	A	G	0.302	0.00464	0.0243	0.849
rs12531458	7	39090698	C	A	0.487	0.0113	0.0224	0.614
rs12702087	7	44812607	A	G	0.437	−0.00242	0.0225	0.914
rs756912	7	71741797	T	C	0.525	0.0203	0.022	0.356
rs11976020	7	72247800	A	G	0.228	0.0067	0.0261	0.798
rs12534506	7	92662327	T	A	0.539	0.0131	0.0226	0.564
rs148490894	7	99531755	G	A	0.0275	0.0856	0.0674	0.204
rs11771168	7	113904061	T	C	0.255	0.0428	0.0263	0.104
rs113520408	7	128402782	A	G	0.278	−0.0582	0.0247	0.0183
rs17167170	7	133302345	G	A	0.205	0.00347	0.0276	0.9
rs320700	7	137049477	A	G	0.641	−0.0159	0.0229	0.486
rs1106761	8	142619234	A	G	0.385	0.0206	0.023	0.371
rs11774212	8	145686505	T	C	0.515	0.00315	0.022	0.886
rs4741343	9	14075095	A	G	0.17	−0.0273	0.0293	0.352
rs4741351	9	14222782	G	A	0.705	−0.039	0.0244	0.111
rs7029201	9	23358081	A	G	0.418	0.00472	0.0224	0.833
rs7033137	9	72055158	G	C	0.26	0.0412	0.0253	0.104
rs17425572	9	88006338	G	A	0.53	−0.00799	0.0221	0.718
rs10821136	9	96238731	T	C	0.33	−0.0115	0.0234	0.625
rs10818606	9	124618386	C	T	0.588	−0.0151	0.0223	0.498
rs10761741	10	65066186	T	G	0.415	0.0302	0.0224	0.177
rs7914680	10	67965010	G	T	0.271	0.0302	0.0252	0.23
rs1925576	10	68689083	G	A	0.451	−0.0128	0.0228	0.575
rs149613931	10	103550281	T	G	0.0564	−0.0516	0.0481	0.284
rs73344830	10	103816828	G	A	0.582	−0.0213	0.0223	0.341
rs61874768	10	103880118	T	G	0.18	−0.0165	0.0292	0.572
rs10786662	10	103989812	C	G	0.582	−0.0126	0.0223	0.573
rs12761761	10	133775375	T	C	0.253	−0.0146	0.0252	0.562
rs7945718	11	12748819	G	A	0.412	0.0346	0.0225	0.123
rs76878669	11	66092567	G	C	0.252	0.072	0.0262	0.00607
rs7948975	11	90424638	C	T	0.398	0.0278	0.0224	0.215
rs111321694	11	110950386	T	C	0.175	−0.0242	0.0292	0.408
rs79925071	11	121998253	T	G	0.571	−0.0136	0.0224	0.545
rs10772644	12	13417617	C	G	0.887	−0.00369	0.0364	0.919
rs7964899	12	14595756	A	G	0.431	−0.0359	0.0222	0.105
rs1389473	12	92154270	A	G	0.388	0.0109	0.0223	0.626
rs10773002	12	123746961	T	A	0.755	0.0173	0.0256	0.501
rs8002014	13	58358159	A	G	0.264	0.0374	0.0248	0.132
rs9556958	13	99100046	T	C	0.519	0.0465	0.0225	0.0388
rs34344888	14	23387585	G	A	0.606	−0.0238	0.0225	0.291
rs1115240	14	27090388	C	G	0.742	−0.0239	0.0251	0.341
rs10483349	14	29629456	G	A	0.184	0.0299	0.0287	0.297
rs58694847	14	84916511	C	G	0.266	0.0107	0.025	0.667
rs1378214	15	47579004	C	T	0.628	0.0795	0.0229	0.000531
rs6493271	15	47613593	C	T	0.179	−0.0475	0.0291	0.102
rs281302	15	47686662	A	G	0.546	−0.0416	0.0227	0.0665
rs12900061	15	66009248	A	G	0.172	0.00978	0.029	0.736
rs4076457	15	78007213	T	C	0.257	−0.00426	0.0253	0.866
rs28420834	15	82513121	G	A	0.573	−0.0238	0.0229	0.298
rs9914544	17	18787828	C	A	0.374	0.0426	0.0227	0.0608
rs9964724	18	35159124	T	C	0.682	−0.00238	0.0237	0.92
rs12956009	18	44768024	C	T	0.426	−0.0195	0.0224	0.384
rs62100765	18	50735418	T	C	0.403	−0.00695	0.0224	0.757
rs1382358	19	13171424	C	T	0.13	−0.0209	0.033	0.527
rs111730030	19	13268826	T	G	0.0578	−0.024	0.0476	0.615
rs12462428	19	16694610	C	T	0.193	−0.0149	0.0278	0.592
rs78387210	20	47823441	T	C	0.0904	−0.0153	0.0393	0.698
rs6065080	20	59832791	C	T	0.645	0.0244	0.023	0.289
rs35532491	22	34329603	T	A	0.101	0.0106	0.0364	0.771
rs7286601	22	51121416	G	T	0.458	−0.00681	0.0222	0.759

*EA, effect allele; NEA, non-effect allele; EAF, effect allele frequency; SE, standard error; Beta is regression coefficients based on effect allele.*

### Pleiotropy Analysis

Evidence from the MR-Egger intercept test supported that these 141 genetic variants showed no significant pleiotropy with intercept = 0.005, *P* = 0.581. Importantly, evidence from the MR-PRESSO global test further highlighted no significant horizontal pleiotropy *P* = 0.375. Hence, these 141 genetic variants could be selected as the effective instrumental variables.

### Univariable Mendelian Randomization Analysis

**FIGURE 1:**
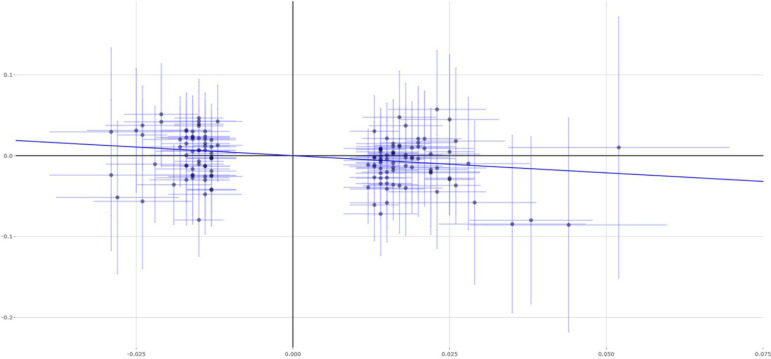
Single estimates about causal association between educational attainment and asthma from MR analysis using IVW method. This scatter plots represent 141 genetic variants associated with educational attainment on *x*-axis and risk of asthma on *y*-axis. Continuous line represents causal effect of educational attainment on risk of asthma. IVW, inverse variance weighted.

**FIGURE 2:**
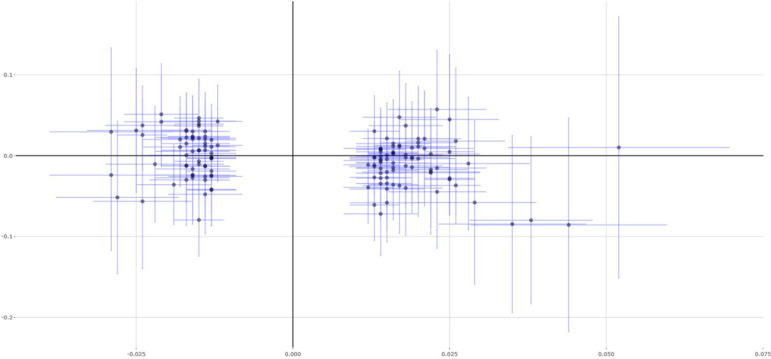
Single estimates about causal association between educational attainment and asthma from MR analysis using weighted median method. This scatter plots represent 141 genetic variants associated with educational attainment on *x*-axis and risk of asthma on *y*-axis. Continuous line represents causal effect of educational attainment on risk of asthma.

**FIGURE 3:**
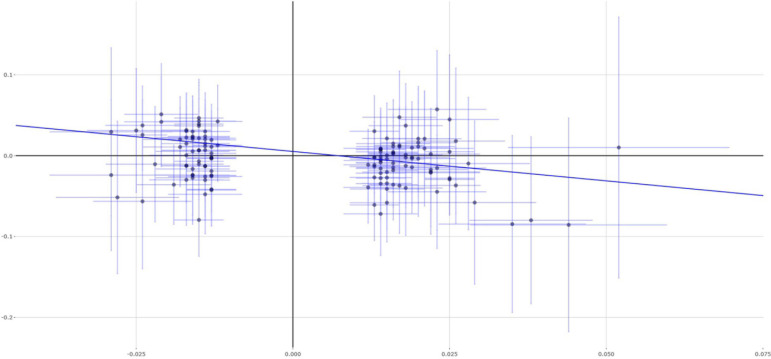
Single estimates about causal association between educational attainment and asthma from MR analysis using MR-Egger method. This scatter plots represent 141 genetic variants associated with educational attainment on *x*-axis and risk of asthma on *y*-axis. Continuous line represents causal effect of educational attainment on risk of asthma.

The main analysis method IVW indicated that each 1 SD increase in educational attainment (3.6 years) could reduce 35% asthma risk (OR = 0.65, 95% CI 0.51–0.85, *P* = 0.001). Importantly, evidence from other MR methods further supported this finding, including weighted median (OR = 0.55, 95% CI 0.38–0.80, *P* = 0.001), MR-Egger (OR = 0.48, 95% CI 0.16–1.46, *P* = 0.198), and MR-PRESSO (OR = 0.65, 95% CI 0.51–0.85, *P* = 0.0015). Figures 1–3 show the individual causal estimates using the IVW method, weighted median, and MR-Egger, respectively. We further conduct a sensitivity analysis using the leave-one-out permutation. The results suggested no single genetic variant to significantly affect the estimates between educational attainment and the risk of asthma.

### Multivariable Mendelian Randomization Analysis

In multivariable MR analysis, we evaluated the effect of cognitive performance on the causal association between educational attainment and the risk of asthma. However, all three multivariable MR analysis methods indicated no significant causal association between educational attainment and the risk of asthma, including multivariable IVW method (OR = 0.64, 95% CI 0.35–1.17, *P* = 0.144), multivariable median-based method (OR = 0.63, 95% CI 0.28–1.42, *P* = 0.265), and multivariable MR-Egger method (OR = 0.32, 95% CI 0.08–1.22, *P* = 0.094). Hence, these findings provide evidence to support that educational attainment protects against asthma risk dependently on cognitive performance.

### Power Analysis

One hundred forty-one educational attainment genetic variants finally selected in our MR analysis explain a total of 3.87% of educational attainment variance. Power analysis using mRnd showed that our MR analysis had 80% power to detect OR of 0.79 or lower per SD increase in educational attainment for the risk of asthma. Meanwhile, our MR analysis has 100% power to detect the OR of 0.65 using IVW, the OR of 0.55 using weighted median, the OR of 0.48 using MR-Egger, and the OR of 0.65 using MR-PRESSO.

## Discussion

Until recently, multiple large-scale GWAS analyses have been conducted to report novel asthma genetic variants (Demenais et al., 2018; Zhu et al., 2018; Shrine et al., 2019; Han et al., 2020). Importantly, these GWAS datasets are publicly available and promote additional analyses, such as MR analysis, to evaluate the causal association between common risk factors and asthma. These risk factors include soluble interleukin-6 receptor level (Rosa et al., 2019; Raita et al., 2021), childhood BMI (Au Yeung et al., 2021a), adult BMI (Granell et al., 2014; Skaaby et al., 2018; Xu et al., 2019; Sun et al., 2020; Au Yeung et al., 2021a), major depressive disorder (Mulugeta et al., 2020), early pubertal maturation (Chen et al., 2020), age at puberty (Minelli et al., 2018), estimated glomerular filtration rate (Park et al., 2021), lifetime smoking (Shen et al., 2020), and linoleic acid (Zhao and Schooling, 2019).

It is reported that socioeconomic status is also a risk factor for asthma (Eagan et al., 2004; Hancox et al., 2004; Kozyrskyj et al., 2010; Brite et al., 2020). In the World Trade Center Health Registry study, Brite et al. (2020) analyzed the data from 30,452 individuals and found that individuals with lower socioeconomic status had worse asthma outcomes. In the Western Australian Pregnancy Cohort (Raine) Study, Kozyrskyj et al. (2010) analyzed the data from 2,868 children and found that children with lower socioeconomic status tended to develop persistent asthma. However, Hancox et al. (2004) reported inconsistent findings in a prospective cohort study including approximately 1,000 individuals in New Zealand. They found no significant between socioeconomic status during childhood and the prevalence of asthma (Hancox et al., 2004). Hence, the causal association between socioeconomic status and asthma remains unclear, which further promotes us to perform an MR analysis using the large-scale GWAS datasets.

Using 162 independent educational attainment genetic variants, we successfully extracted the summary association results of 141 unique genetic variants from the asthma GWAS dataset. The pleiotropy analysis indicated these genetic variants to be effective instruments. MR analysis showed each 1 SD increase in educational attainment (4.2 years) reduced 35% asthma risk (OR = 0.65, 95% CI 0.51–0.85, *P* = 0.001) using IVW. Importantly, other additional analysis methods and sensitivity methods supported this finding. However, multivariable MR analysis showed that educational attainment protected against asthma risk dependently on cognitive performance.

Until now, univariable and multivariable MR studies have evaluated the association of educational attainment and/or cognitive performance on other human complex diseases or phenotypes. Wang et al. (2021) conducted a two-sample univariable and multivariable MR to evaluate the causal effects of educational attainment and cognition on the risk of epilepsy. Using univariable MR analysis, they found that both educational attainment and cognitive performance could reduce the risk of epilepsy (Wang et al., 2021). Using multivariable MR analysis, they found that only educational attainment protected against epilepsy independent of cognitive performance (Wang et al., 2021).

Gill et al. (2019) conducted a two-sample univariable MR to evaluate the effect of education and cognitive performance, respectively, on the risk of coronary heart disease and ischemic stroke. Meanwhile, they performed a multivariable MR to adjust for the effects of cognitive performance and education, respectively (Gill et al., 2019). Using univariable MR analysis, they found a causal association between high education and reduced risk of coronary heart disease and stroke (Gill et al., 2019). Meanwhile, they also found that high cognitive performance could also reduce the risk of coronary heart disease but not stroke (Gill et al., 2019). Using multivariable MR analysis, they found that education could protect against coronary heart disease and stroke independent of cognitive function (Gill et al., 2019). However, the cognitive performance had no causal association with coronary heart disease or stroke by adjusting for education (Gill et al., 2019). Carter et al. (2019) found that BMI, systolic blood pressure, and smoking behavior could mediate the protective role of education on the risk of cardiovascular outcomes, including coronary heart disease, stroke, myocardial infarction, and cardiovascular disease (all subtypes; all measured in OR).

Liang et al. (2021) identified that educational attainment protected against type 2 diabetes independently of cognitive performance. Rosoff et al. (2020) found that educational attainment could reduce the risk of suicide attempts in individuals with and without psychiatric disorders independent of cognition. Zhang et al. (2020) found that high educational attainment, but not cognitive performance, was causally associated with a reduced risk of amyotrophic lateral sclerosis. Meanwhile, MR studies have found that increased education could reduce the risk of ischemic stroke (Gill et al., 2019; Xiuyun et al., 2020; Harshfield et al., 2021) and Alzheimer’s disease (Larsson et al., 2017; Anderson et al., 2020; Andrews et al., 2021; Zhuang et al., 2021a). Anderson et al. (2020) recently examined whether educational attainment and cognitive performance had causal effects on the risk of Alzheimer’s disease, independently of each other. They found that educational attainment affected the risk of Alzheimer’s disease dependently of cognitive performance (Anderson et al., 2020). However, cognitive performance affected the risk of Alzheimer’s disease independently of educational attainment (Anderson et al., 2020).

Hence, all these findings discussed earlier indicated that educational attainment had causal effects on the risk of epilepsy (Wang et al., 2021), coronary heart disease (Gill et al., 2019), stroke (Gill et al., 2019), type 2 diabetes (Liang et al., 2021), and suicide attempt (Rosoff et al., 2020), independently of cognitive performance. However, the causal effect of educational attainment on the risk of Alzheimer’s disease may be mediated by cognitive performance (Anderson et al., 2020). Our findings are consistent with recent MR findings in other human complex diseases or phenotypes.

Since 2018, multiple large-scale asthma GWAS datasets have been reported, as described in the *Introduction*. Here, we only selected the large-scale asthma GWAS dataset in 30,810 individuals of European ancestry from Shrine et al. (2019). In brief, these GWAS samples are from GASP, U-BIOPRED, and the UK Biobank (Shrine et al., 2019). In 2018, TAGC examined the common asthma variants by a meta-analysis of worldwide asthma GWAS datasets, including 23,948 asthma cases and 118,538 controls (Demenais et al., 2018). However, all these individuals are from ethnically diverse populations, including European ancestry, African ancestry, Japanese ancestry, and Latino ancestry (Demenais et al., 2018). It is known that all these selected educational attainment genetic variants are from the large-scale GWAS dataset in individuals of European descent (*n* = 405,072) (Okbay et al., 2016). Hence, we did not select the asthma GWAS dataset from TAGC in our MR analysis (Demenais et al., 2018). Zhu et al. (2018) conducted a genome-wide cross-trait analysis to investigate the shared genetic etiology in asthma and allergic diseases by analyzing large-scale GWAS datasets from the UK Biobank, including 25,685 allergic diseases subjects, 14,085 asthma subjects, and 76,768 controls. Hence, both Shrine et al. (2019) and Zhu et al. (2018) have used the UK Biobank samples. Hence, we did not select the asthma GWAS dataset from Zhu et al. (2018) in our MR analysis. Han et al. (2020) conducted a GWAS using 64,538 asthma cases and 329,321 controls from UK Biobank and then performed a meta-analysis using the UK Biobank and the TAGC datasets. However, they did provide the effect size and the corresponding standard error for each variant in the GWAS summary dataset (Han et al., 2020). Importantly, there is a sample overlap in both studies from Shrine et al. (2019) and Han et al. (2020), as both shared the UK Biobank samples. Hence, we did not select the GWAS dataset from the UK Biobank or the GWAS dataset from the meta-analysis of the UK Biobank and TAGC in our MR analysis (Han et al., 2020).

Meanwhile, our MR analysis still has some limitations. First, our findings are based on the educational attainment GWAS dataset and asthma GWAS dataset in individuals of European ancestry (Okbay et al., 2016; Shrine et al., 2019). It remains unclear about the causal association between educational attainment and asthma in other ancestries. Hence, replication MR studies are required to investigate our findings in the future. Second, both the educational attainment GWAS dataset and asthma GWAS dataset include the samples from the UK Biobank (Okbay et al., 2016; Shrine et al., 2019). In brief, the replication stage in the educational attainment GWAS dataset included 111,349 individuals from the UK Biobank (Okbay et al., 2016). The asthma GWAS dataset included 2,996 asthma cases from the UK Biobank and 25,600 controls from the UK Biobank (Shrine et al., 2019). Hence, the educational attainment GWAS dataset and the asthma GWAS dataset may not be independent. Hence, independent GWAS datasets are also required to evaluate our findings further.

In summary, we highlight the protective role of educational attainment against asthma with 100% statistical power using univariable MR analysis. Meanwhile, we provide evidence to support that educational attainment protects against asthma risk dependently on cognitive performance using multivariable MR analysis. Our findings may have public health applications and deserve further investigation.

## Data Availability Statement

The original contributions presented in the study are included in the article/Supplementary Material, further inquiries can be directed to the corresponding author/s.

## Author Contributions

YL and BY designed the project and analyzed the data. All authors wrote the first draft of the manuscript, revised, and approved the final manuscript.

## Conflict of Interest

The authors declare that the research was conducted in the absence of any commercial or financial relationships that could be construed as a potential conflict of interest.

## Publisher’s Note

All claims expressed in this article are solely those of the authors and do not necessarily represent those of their affiliated organizations, or those of the publisher, the editors and the reviewers. Any product that may be evaluated in this article, or claim that may be made by its manufacturer, is not guaranteed or endorsed by the publisher.
